# Independent and interactive effect of type 2 diabetes and hypertension on memory functions in middle aged adults

**DOI:** 10.1186/s12902-023-01308-3

**Published:** 2023-03-10

**Authors:** Kinga Kálcza Jánosi, Andrea Lukács

**Affiliations:** 1 Faculty of Psychology and Educational Sciences, Babes-Bolyai University, 7, Sindicatelor Street, 400604 Cluj-Napoca-Napoca, Romania; 2 Faculty of Health Sciences, University of Miskolc, 3515 Miskolc-Egyetemváros, Hungary

**Keywords:** Attention and concentration, Delayed memory, Hypertension, Type 2 diabetes, Verbal memory, Visual memory

## Abstract

**Background:**

The study distinguishes the effect of type 2 diabetes and hypertension on cognitive functions when the two diseases are alone or when they occur together, compared to healthy individuals.

**Methods:**

A total of 143 middle-aged adults were screened using the Wechsler Memory Scale – Revised psychometric test (verbal memory, visual memory, attention/concentration and delayed memory). Participants were divided into four groups based on their diseases: patients with type 2 diabetes (36), patients with hypertension (30), patients having both diseases (33), and healthy controls (44).

**Results:**

This study found no differences among investigated groups in verbal and visual memory, however, hypertension and both-disease group performed unfavorably compared to patients with diabetes and to healthy individuals in attention/concentration and delayed memory.

**Conclusions:**

The findings of this study suggest that there is a relationship between hypertension and cognitive dysfunction, whereas type 2 diabetes without consequences was not proved to have an association with cognitive decline in middle-aged people.

## Background

Diabetes and hypertension are a worldwide epidemic, resulting in millions of deaths each year. These two diseases often occur together and exacerbate each other’s symptoms, and may share common pathways such as insulin resistance, inflammation, obesity and oxidative stress [1]. The coexistence of type 2 diabetes (T2D) and hypertension is increasing in the general population. Hypertension is disproportionately higher in patients with diabetes. Having one of the two conditions increases the risk of developing the other disease by 1.5–2.0 times [2]. The brain is one of the main target organs affected by high glucose level and hypertension, and both conditions might be associated with a wide variety of cognitive impairments. Especially type 2 diabetes and hypertension frequently coexist, and their combination may provide additive increases in the risk impairment of cognitive processes [3].

Cognitive disorders play an important role in diabetes and hypertension for two reasons: first, cognitive disorders are complications associated with the diseases and may indicate the patient has inadequate glucose metabolism and/or high blood pressure. Secondly, the cognitive dysfunctions have a major impact on the self-management of the disease and can influence the quality and progression of the diseases in the future. Awad’s et al. [4] proposed cognitive functions are more affected in patients with T2D, who have various associated complications such as hypertension. Other comorbid illnesses such as clusters of comorbidities that combine with hypertension may have a greater cognitive impact. Some studies suggest hypertension and diabetes, when combined, increase the risk of cognitive impairment [5, 6]. The main question, which has not yet been fully clarified, is whether having T2D combined with hypertension increases the risk of cognitive decline. Previous studies mainly evaluated older individuals with T2D [7], and knowledge regarding middle-aged people is scarce. To fill this gap, this study aims to answer the question of whether middle-aged individuals with independent T2D (without hypertension), independent hypertension (without T2D), or comorbid T2D and hypertension have some dysfunction in memory components (verbal memory, visual memory, attention and concentration, delayed memory) specific to the selected risk groups, compared to healthy individuals.

## Methods

### Study design, ethics, and participants

A cross-sectional quantitative survey was performed in Harghita County (Transylvania, Romania). The patients were recruited from three randomly chosen settlements (Joseni, Ciumani and Lăzarea) with the help of general practitioners of these villages. All of the patients were informed about the purpose of the study and the voluntary nature of the participation. Participants gave written informed consent before taking part in the study. The protocol of the study was approved by the Ethics Committee of the University of Babeș-Bolyai (RO) and by the Code of Deontology for the professions of psychologists, elaborated by the Romanian College of Psychologists. Cognitive functions were assessed independently for each participant by accredited clinical psychologists. Inclusion criteria were: 1) ages between 35 and 65 years [8, 9], 2) diagnosed with T2D or hypertension or both (comorbid T2D and hypertension) according to the standards of the American Diabetes Association (2020) [10] and according to the standards of the American College of Cardiology and American Heart Association guidelines [11], respectively, and 3) diabetes and/or hypertension duration at least 5 years. From this study, patients were excluded with 1) any medical illnesses other than T2D, dyslipidemia, hypertension and obesity, 2) a history of hypoglycemic coma or complications of diabetes, 3) primary neurological condition as history of transient ischemic attacks, cerebrovascular stroke or epilepsy or psychiatric disease, 4) previous serious head injury, 5) any sensory or motor disorder that would preclude psychological testing (including blindness), 6) regular treatment with any medications known to have psychoactive effect, and 7) drug or alcohol abuse. The control group was made up of people who applied at the invitation of the municipality's management and did not have a chronic illness. Their health status was checked at the health clinic of the municipality.

The patients were divided into three groups: a group with T2D, a group with hypertension and a group with both diseases. The control group was specifically recruited from the settlement where the patients were registered.

### Measurements

#### Demographics

Patients provided data about their gender, age, and education duration in years, as well as the duration of the disease.

#### Clinical parameters

Blood pressure was measured on the left arm in sitting posture during the visit by the physician using an aneroid sphygmomanometer with a stethoscope.

Glycemic control was explained by HbA1c (the gold standard measurement of glycemic control) [10]. The last three-month HbA1c value was extracted from the medical records of patients with diabetes, or was measured from a vein during the routine visit.

#### Cognitive measures

Cognitive functions were assessed using the Wechsler Memory Scale – Revised (WMS-R) [12]. The test was administered during a routine visit to the general practitioner and it required around 1 h to complete. The WMS-R is a neuropsychological test designed to measure different memory functions such as verbal, visual memory, attention/concentration and delayed memory. The psychometric characteristics of the WMS-R are evaluated and related to its clinical utility in the Romanian population [13, 14]. The weighted scores (weighted raw score summaries) were calculated according to the scoring system from in the WMS-R administrative and scoring manual, with a higher score indicating better functioning.

### Statistical analysis

#### Data analysis

For data analysis SPSS (Statistical Package for the Social Sciences) version 25.0 was used (SPSS, Version 25, IBM Corporation, Armonk, NY). All *p*-values were two-tailed at the significance level of 0.05. In the first stage, the Z-score between -3 and 3 method of outlier detection was performed and removed from the database. Violations of the normality assumption were checked using Shapiro–Wilk’s test. As a result, the memory variables were transformed by square-root transformation (moderately positively and negatively skewed data). The differences between investigated groups on demographic and clinical information were tested with analysis of variance (one-way ANOVA) for continuous outcomes and Pearson’s chi-square test for dichotomous outcomes. Comparison of memory scores between groups was measured using multiple analysis of covariance (MANCOVA) with follow-up univariate ANOVAs and the Bonferroni post-hoc test. Age, sex and education duration were controlled, age and education as covariates and also gender as a factor. We checked the assumptions that are required for MANCOVA, assumption of independence, assumption of normality, assumption of homogeneity of variance and assumption of absence of multicollinearity.

#### Power and sample size

A post hoc power analysis was conducted using the software package GPower. The recommended effect sizes used for this assessment were as follows: small (*f *^*2*^ = 0.02), medium (*f *^*2*^ = 0.15), and large (*f *^*2*^ = 0.35) [15]. The alpha level used for this analysis was *p* ≤ 0.05. The power of the study was high to detect the main outcome of interest. The post hoc analysis revealed that the statistical power for this study was 99% (0.99) for detecting medium effect (0.12) for statistical comparisons (MANCOVA, *N* = 143, 4 groups, 4 response variables).

## Results

### Patients and clinical variables

The initial group included 157 individuals but identifying outliers eventually resulted in 143 eligible persons. The control group consisted of 44 healthy adults. Participants were homogeneous by age, sex and education duration: there were no significant differences among the four investigated groups in age, gender or education duration, nor in clinical characteristics such as diabetes duration and glycemic control between groups with T2D and presenting both diseases. No significant differences were observed in duration of hypertension or systolic/diastolic blood pressure between the groups with hypertension and with both diseases. Characteristics of the participants stratified by disease are displayed in Table 1.

**Table 1:** Characteristics of the participants stratified by disease (*N* = 143)

Mean, Standard Deviation (SD)	Healthy group (*n* = 44)	Group with T2D (*n* = 36)	Group with HTN (*n* = 30)	Group with both diseases (*n* = 33)	*F(df)*	*X*^*2*^*(df)*	*p*
Age (years)	46.40 (4.09)	45.91 (5.16)	45.83 (4.83)	46.81 (5.48)	0.295_(3,139)_	-	0.829
Male sex, *n* (%)	23 (52.3%)	17 (47.2%)	14 (46.7%)	17 (51.1%)	-	.359_(3)_	0.949
Education (years)	12.72 (1.70)	12.02 (1.69)	12.66 (1.98)	12.36 (2.26)	1.05_(3,139)_	-	0.371
HbA_1c_	-	7.46 (0.54)	-	7.61 (0.55)	1.35_(1,67)_	-	0.248
Diabetes duration (years)	-	7.66 (2.44)	-	8.54 (2.57)	2.11_(1,67)_	-	0.151
Treatment regime (%) (frequency)	-		-		-	-	-
Insulin		11 (4)		17 (6)			
OGLM		57 (21)		61 (20)			
Diet		32 (11)		22 (7)			
Antihypertensive med (%) (frequency)	-	-	34 (10)	48 (16)	-	-	-
Hypertension duration (years)	-	-	8.96 (2.41)	9.81 (2.93)	1.56_(1,61)_	-	0.134
SBP 24 h (mmHg)	-	-	144.47 (8.11)	147.12 (7.41)	1.84_(1,61)_	-	0.180
DBP 24 h (mmHg)	-	-	90.73 (5.08)	91.39 (4.19)	.319_(1,61)_	-	0.574

*T2D* Type 2 diabetes, *HTN* Hypertension, *HbA*_*1c*_ Glycosylated hemoglobin, *OGLM* Oral glucose-lowering medication, *SBP* Systolic blood pressure, *DBP* Diastolic blood pressure

### Neuropsychological functions (verbal memory, visual memory, attention and concentration, delayed memory)

There were statistically significant differences in memory functions based on disease, F_(12, 344)_ = 4.240, p ≤ 0.01; Wilk's Λ = 0.694, partial η^2^ = 0.114, approximately 11.4% of multivariate variance of the dependent variables is associated with the group factor (MANCOVA) after controlling for age (F_(4, 130)_ = 1.220, *p* > 0.05), education duration (F_(4, 130)_ = 5.914, p ≤ 0.01) and sex (F_(4, 130)_ = 0.052, *p* > 0.05).

**Fig. 1:**
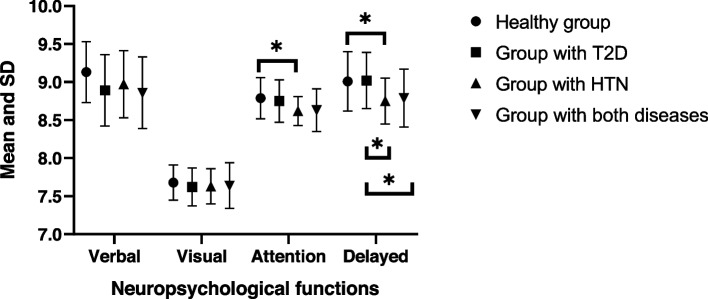
Raw scores with mean and SD of the neuropsychological functions (*N* = 143). T2D—type 2 diabetes, HTN—hypertension, *p ≤ 0.05

Follow-up univariate ANOVAs indicate significant differences between the groups in attention and concentration (F_(3, 133)_ = 4.026, *p* ≤ 0.01; partial η^2^ = 0.083) and delayed memory (F_(3, 133)_ = 5.665, *p* ≤ 0.01; partial η^2^ = 0.113), meaning that the disease has a statistically significant effect on these cognitive functions. Mean scores of attention and concentration were significantly different between the control group (M = 8.79, SD = 0.27) and group with hypertension (M = 8.62, SD = 0.19; *p* ≤ 0.05), and the status of the control group was better than that of the other two groups. For delayed memory, the control group (M = 9.01, SD = 0.39) showed a significantly better status than the group with hypertension (M = 8.75, SD = 0.30; *p* ≤ 0.05), while the group with T2D (M = 9.02, SD = 0.37) had a better status than the group with hypertension (M = 8.75, SD = 0.30; *p* ≤ 0.05) and the group with both diseases (M = 8.79, SD = 0.38; *p* ≤ 0.05). (Table 2) Raw scores of the neuropsychological functions with mean and SD are displayed in Fig. 1.

**Table 2:** Neuropsychological functions with participants stratified by disease (*N* = 143)

Mean (SD) memory	Healthy group (*n* = 44)	Group with T2D (*n* = 36)	Group with HTN (*n* = 30)	Group with both diseases (*n* = 33)	F_(df)_	*p*	partial η^2^
Verbal	9.13 (.40)	8.89 (.47)	8.97 (.44)	8.86 (.47)	2.515_(3.133)_	0.061	0.054
Visual	7.68 (.23)	7.62 (.25)	7.63 (.23)	7.64 (.30)	.331_(3.133)_	0.803	0.007
Attention and concentration	8.79 (.27)	8.75 (.28)	8.62 (.19)	8.63 (.28)	4.026_(3.133)_	**0.009**	0.083
Delayed	9.01 (.39)	9.02 (.37)	8.75 (.30)	8.79 (.38)	5.665_(3.133)_	**0.001**	0.113

*T2D* Type 2 diabetes, *HTN* Hypertension

## Discussion

The present study investigated whether there are any differences in memory functioning among adults with independent T2D (without hypertension), independent hypertension (without T2D), with comorbid hypertension and T2D, and healthy individuals. The literature is not clear on whether diabetes affects premature cognitive decline [16–19]. In our study, we found no differences between groups in the capacity to remember what the individual had previously learned (read or heard) and recall when needed (verbal memory). Similarly, no significant differences were found in visual memory between the investigated groups. Individuals had similar capacity to remember or recall information that had been previously viewed. The findings of our study cannot confirm that cognitive functions such as verbal memory, visual memory, attention and concentration, as well as delayed memory have a relationship with the presence of T2D, however, for the latter two, differences were found among groups. Groups with hypertension and with both diseases displayed unfavorable results compared to the group with diabetes and the healthy group in the functions of attention and concentration and in delayed memory. Moran et al. [20] also failed to prove a direct effect of T2D on cognitive function. Demakakos et al. [21] examined the combined effect of diabetes and elevated depressive symptoms and found they significantly accelerate cognitive decline over time, especially in people aged 50–64, but do not accelerate cognitive decline separately. The results of our study suggest hypertension has a negative impact on cognitive functions, and co-occurrence with diabetes does not exacerbate this. Muela et al. [22] evaluated patients with hypertension compared to healthy controls and found poorer cognitive performance in almost all cognitive tests. Only a few studies have explicitly studied the cognitive impacts of comorbid hypertension and T2D, reporting that the two conditions may interact to increase the risk of cognitive impairment [23, 24] or may not [25, 26]. We found no significant differences in cognitive components between the people with diabetes and the healthy adults, which indicate that cognitive deficits appear more pronounced in individuals who have hypertension, with or without T2D. It should be mentioned that we cannot unequivocally determine the direction of the association between cognitive function and hypertension and T2D. The relationship between cognitive changes and health outcomes is bidirectional. Medical aspects of hypertension and T2D can negatively affect cognitive functioning, while deterioration of cognitive functioning can negatively influence the self-management of disease. Those who are cognitively impaired have poorer command of the skills necessary to manage their chronic diseases. It is worth noting that our middle-aged patients with T2D had no vascular complications that might have resulted in cognitive impairment. Both T2D and hypertension are chronic psychosomatic diseases and the resulting complications can affect the entire body, requiring specific attention to both the cognitive and the somatic components of the diseases [27, 28].

Limitations of the study include a relatively small proportion of participants from one county which precludes generalizability. The cross-sectional design does not allow us to infer causality. Smoking status has not been investigated, which could help to understand its weight in the genesis of the change in memory. Dyslipidaemia was not assessed, and could play a possible role in the impaired memory pattern.

## Conclusion

Our results indicate that there is an association between hypertension and cognitive impairments in middle-aged patients with T2D, whereas this association is not proved between complication-free T2D and cognitive function. Regarding verbal and visual memory, no significant differences were found among groups, so it seems likely that these memory dimensions are not influenced by the investigated diseases. Regarding attention, concentration and delayed memory, the cognitive deficits are pronounced when hypertension is present. There is no evidence that co-occurrence of T2D amplifies the effect of hypertension on cognitive decline.

## Data Availability

Data are available upon reasonable request. To access the required data, the researchers can contact Andrea Lukács PhD, Email: andrea.lukacs8080@gmail.com

## Abbreviations

RO: Romania
T2D: Type 2 diabetes
WMS-R: Wechsler Memory Scale – Revised
HbA1c: Hemoglobin A1c
